# Atypical Trigeminal Neuralgia Exacerbation Following Hypoglossal Nerve Stimulator Implantation: A Case Report

**DOI:** 10.1002/oto2.60

**Published:** 2023-07-22

**Authors:** Jonathan L. Vincze, Megan E. Falls, Noah P. Parker

**Affiliations:** ^1^ Marian University College of Osteopathic Medicine Indianapolis Indiana USA; ^2^ Department of Otolaryngology–Head and Neck Surgery Indiana University School of Medicine Indianapolis Indiana USA

**Keywords:** atypical trigeminal neuralgia, concomitant continuous pain, Inspire stimulator, Type 2 trigeminal neuralgia, upper airway stimulator

Trigeminal neuralgia (TN) is defined as paroxysmal, sharp pain within the trigeminal nerve (CNV) distribution lasting up to 2 minutes.^1^ In contrast, atypical trigeminal neuralgia (ATN) is a rare, chronic disorder characterized by burning pain and paresthesia within the CNV distribution. While TN and ATN were previously thought to be idiopathic in origin, recent evidence suggests that many cases are due to nerve compression leading to hyperexcitability.^1^, ^2^ ATN is characteristically more refractory to treatment than TN, suggesting that these conditions may have a different pathophysiology.^1^, ^2^, ^3^ Pharmacotherapy is the primary treatment for both conditions.^3^ However, for patients who fail or cannot tolerate medical management, gamma knife radiation (GKR) is an accepted treatment option.^2^

Hypoglossal nerve stimulation (HNS) is a modality of obstructive sleep apnea (OSA) treatment wherein an Inspire device (Inspire Medical Systems) is surgically implanted to stimulate the extension of the tongue via branches of the hypoglossal nerve (CNXII) during inspiration. This keeps the pharynx from collapsing and, thus, improves OSA.^4^ We present the first case report of a patient who experienced exacerbation of ATN symptoms with HNS device usage. This case report was deemed exempt by the Indiana University institutional review board.

A 63‐year‐old male with body mass index 27.2 presented for evaluation of OSA with apnea–hypopnea index of 22.9/hour and positive airway pressure intolerance. His medical history included right‐sided TN following lightning strike 35 years ago and refractory to medical therapy. He underwent GKR 3 years prior, with improvement in TN symptoms. He was deemed an appropriate candidate for HNS therapy. Placement of and recovery from HNS implantation was without incident.

After device activation, the patient reported reduced snoring and improved daytime energy, but increased right facial discomfort. Over the subsequent weeks, he noted progressive aching right facial pain, small shocks in the corner of the eye, worm‐like crawling in his cheek, and hypersensitivity and numbness in the lower lip. Magnetic resonance imaging (MRI) was notable only for asymmetric enhancement of the right CNV in Meckel's cave, consistent with GKR changes.

The HNS device was discontinued for 4 weeks, then retried with lower‐voltage settings. His discomfort initially improved, but reintroduction precipitated symptoms. It was subsequently discovered that prior to HNS implantation, he had reported subtle, but increasing right‐sided facial pain. Following several conversations between sleep surgery, sleep medicine, neurology, and neurosurgery, it was concluded that he developed ATN prior to device placement and, unfortunately, usage led to symptom exacerbation. Advanced titration of the device was suggested to see if altered electrode configurations or pulse width or rate could help, but the patient declined. Patient desired explantation to eliminate MRI restrictions, which was performed without issue. He was ultimately treated medically for ATN with improved symptoms.

## Discussion

There is no known physiologic connection between CNXII and CNV, which controls facial sensation and provides motor branches to the muscles of mastication, mylohyoid, tensor veli palatini, and digastric muscles. Thus, a CNXII stimulator would not be expected to exacerbate trigeminal pain. Despite this, HNS device usage clinically exacerbated symptoms and disuse improved them. TN and ATN have multiple triggers, including touching the face, eating, and talking.^1^, ^2^ In retrospect, we hypothesize that if such activities can exacerbate symptoms, so could stimulation of the genioglossus and geniohyoid muscles, not to mention the potential recruitment of the palate via palatoglossus. Although unusual, stimulation of this musculature could theoretically have transmitted to the mylohyoid and tensor veli palatini.

Multidisciplinary collaboration was key to understanding the patient's underlying facial pain disorder and the contribution of the HNS device.^3^ Should future HNS programs encounter a similar scenario, medical therapy is an excellent approach, but early advanced device titration with awake endoscopy or placebo activation are also reasonable. While not a contraindication to HNS, patients with TN or ATN should be counseled that their symptoms may change with device use. Meanwhile, the interplay between HNS and pre‐existent neuralgias is worthy of future study.

## Conclusion

There is no neurophysiologic connection between the trigeminal and hypoglossal nerve, yet this patient's ATN was exacerbated by HNS use and resolved only with disuse. Further evaluation of facial pain in patients treated with HNS is warranted, as is patient counseling in this regard.

## Author Contributions

**Jonathan L. Vincze**, manuscript writing and editing, presentation of research; **Megan E. Falls**, manuscript writing and editing, conceptualization; **Noah P. Parker**, manuscript editing, conceptualization.

## Disclosures

### Competing interests

Dr Parker is on a physician advisory council for Inspire. The other authors have no commercial associations or financial disclosures that might pose or create a conflict of interest.

### Funding source

No external funding was used for this study.
